# The Role of Poverty and Racial Discrimination in Exacerbating the Health Consequences of COVID-19

**DOI:** 10.1016/j.lana.2021.100178

**Published:** 2022-01-07

**Authors:** Zachary Parolin, Emma K. Lee

**Affiliations:** a Department of Social and Political Sciences, Bocconi University, Milan, Italy; b Columbia University Center on Poverty and Social Policy, New York, NY, USA

**Keywords:** COVID-19, Poverty, Public health, Health disparities, Racial discrimination

## Abstract

There were more than 800,000 confirmed coronavirus disease 2019 (COVID-19) deaths in the United States (U.S) by the end of 2021. The health consequences of COVID-19, however, have not affected all residents equally. In this review, we synthesize recent evidence suggesting that high levels of poverty in the U.S. compared to other high-income countries, as well as historic and ongoing racial/ethnic discrimination, have exacerbated the health consequences of COVID-19, particularly for racial/ethnic minorities. We discuss four mechanisms through which poverty and discrimination affect COVID-19-related health consequences: greater pre-existing health challenges, reduced access to healthcare, lower-quality neighbourhood and housing conditions, and unequal exposure to high-risk occupations. Evidence suggests that economic and policy institutions that contributed to higher pre-pandemic poverty rates in the U.S., particularly among racial/ethnic minorities, have been central determinants of unequal health outcomes during the COVID-19 pandemic.

## Poverty and Racial Discrimination

The coronavirus disease 2019 (COVID-19) is a global pandemic caused by the spread of severe acute respiratory syndrome coronavirus 2. Within the U.S., the health consequences of the COVID-19 pandemic have not been shared evenly. In this review, we synthesize recent evidence suggesting that poverty and racial/ethnic discrimination have exacerbated the health consequences of the ongoing pandemic.

Poverty and racial/ethnic discrimination are closely intertwined. Racial biases and racialized perceptions of who receives social assistance benefits, for example, have influenced social policy and labour market institutions in the U.S.^1^, ^2^ The influence of discrimination on these laws, policies, and practices have led to higher poverty rates, particularly for racial/ethnic minorities.^1^ Moreover, racial/ethnic discrimination has been shown to contribute to disparities in education and employment outcomes, two factors that directly affect poverty.^2^ In turn, the U.S. had a high poverty rate relative to other high-income countries prior to the pandemic.^3^ Within the U.S., 18.3% of Black residents and 18.9% of Hispanic residents in the U.S. were in poverty, compared to 8.2% of non-Hispanic White residents, according to the Census Bureau's Supplemental Poverty Measure in 2019.^4^ Put differently, evidence suggests that racial/ethnic minorities are more likely to be in poverty, while historic and ongoing racial discrimination have weakened the welfare state and labour market institutions that could further reduce poverty.^1^, ^2^, ^3^, ^4^ Thus, this review understands poverty and racial/ethnic discrimination as closely connected phenomena that may influence the health consequences of the pandemic.

Specifically, we discuss four mechanisms through which poverty and discrimination affect COVID-related health consequences: greater pre-existing health challenges, reduced access to healthcare, lower-quality neighbourhood and housing conditions, and unequal reliance on employment in high-risk occupations. We first review evidence of unequal health outcomes. Afterward, we discuss these four mechanisms in detail.

## Poverty, Racial Discrimination, and COVID-19 Health Outcomes

The health consequences of the COVID-19 pandemic have not been shared evenly. By early May 2020, U.S. counties with higher poverty rates had higher COVID-19 cases and related death rates, on average.^5^ When considering COVID-19 related hospitalizations, lower-income individuals are more likely to be admitted to the Intensive Care Unit (ICU) or to require intermittent mandatory ventilation.^6^ Additionally, lower income U.S. counties experienced more COVID-19 deaths than higher income counties after the first few months of the pandemic (albeit fewer cases, likely due to unequal access to testing as we discuss later).^7^

Racial/ethnic minorities are at increased vulnerability for COVID-19 when considering both infection and mortality rates. A cross-sectional study of 1,000 children, who were tested for COVID-19 at the same testing center, showed that racial/ethnic minority and low-income children had higher COVID-19 positivity rates compared to non-Hispanic White and higher-income children.^8^ Various other studies present similar findings, where Black and Hispanic individuals have higher case and mortality rates for COVID-19, as well as more severe clinical outcomes, when compared to White counterparts.^9^, ^10^, ^11^ Similarly, American Indian and Alaska Native (AIAN) individuals are 1.7 times more likely to be infected with COVID-19, 3.4 times more likely to be hospitalized, and 2.4 times more likely to suffer from COVID-19-associated mortality compared to non-Hispanic White individuals.^12^ There were mixed results when considering COVID-19 case and mortality rate disparities among Asian individuals, with some studies showing significant racial/ethnic disadvantages and others indicating no significant disadvantage.^10^^,^^12^ Data from the Centers for Disease Control (CDC) show similar results, with AIAN, Black, and Hispanic persons experiencing greater COVID-19 related cases, hospitalizations, and deaths.^12^

As a consequence of racial/ethnic inequality in COVID-19 cases and related mortality rates, the reported life expectancies of racial/ethnic minority populations have greatly decreased relative to the life expectancy of White individuals in America. For example, it is estimated that Black and Hispanic populations experienced a 3 to 4 times greater decrease in life expectancy due to the COVID-19 pandemic compared to White individuals.^13^

**Figure 1:**
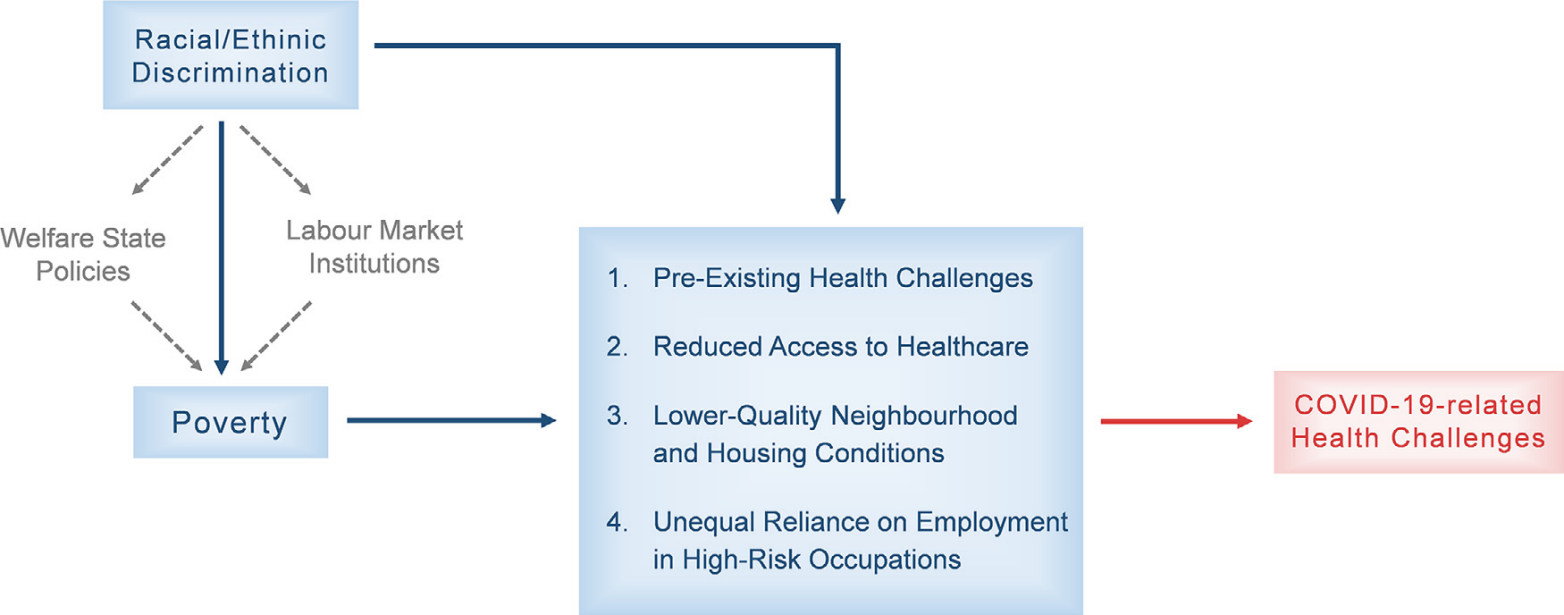
Mechanisms through which poverty and racial/ethnic discrimination affect COVID-19 health disparities

Evidence points to four primary mechanisms through which poverty and discrimination contribute to COVID-related health disparities. Figure 1 summarizes these mechanisms.

The first mechanism relates to **greater pre-existing health challenges**. Certain underlying health conditions make individuals more vulnerable to COVID-19-related hospitalization, intensive care, and mortality. Individuals of lower income and racial/ethnic minority groups disproportionately have underlying medical conditions such as cancer, chronic lung disease, diabetes, heart conditions, and HIV, all of which are influenced by structural and interpersonal discrimination closely embedded in many social determinants of health.^14^ The CDC identified these underlying health conditions as having the potential to exacerbate the medical impact of COVID-19.^15^ Moreover, evidence suggests that poverty status is closely associated with health.^16^ Consider, for example, that 35% of non-elderly adults with an annual household income less than $15,000 are at higher risk for serious illness related to COVID-19, compared to only 16% of non-elderly adults with a household income greater than $50,000.^17^ When considering race/ethnicity, minority populations are more likely to have an underlying health condition than White individuals, in part due to their greater exposure to poverty.^17^

These pre-existing health disparities are closely attached to the second mechanism: **reduced access to healthcare.** Lower-income individuals are less likely to have health insurance than higher income individuals.^18^ In turn, healthcare access varies by race/ethnicity: 21.7% of AIAN, 20.0% of Hispanic, 12.7% of Native Hawaiian and Other Pacific Islander (NHOPI), and 11.4% of Black individuals do not have health insurance, compared to 7.5% of non-Hispanic White and 6.8% of Asian individuals.^19^ When specifically considering individuals with underlying health conditions at high-risk for severe COVID-19 health outcomes, 18.2 million of these individuals are uninsured or underinsured (defined as skipping a visit to the doctor in the past year due to monetary expense).^20^ A disproportionate number of these individuals are Black, AIAN, or non-White and non-Asian.^20^

The high cost of health insurance, driven in part by state policy decisions that reduce access to healthcare for lower-income individuals, helps to explain the economic gaps in health coverage. Specifically, lower income and racial/ethnic minority individuals are more likely to experience barriers to adequate healthcare due to monetary expense, bias related to socioeconomic status, historical (and ongoing) mistrust in the healthcare system, language barriers, and state or federal policy decisions. In early 2020, for example, 13 U.S. states refused to adopt the Affordable Care Act Medicaid Expansion, making it more difficult for low-income residents in these areas to access adequate healthcare.^21^ Moreover, despite the presence of the Indian Health Service (IHS), a federal government division that provides healthcare to AIAN people, there remain accessibility barriers and qualification restrictions, in addition to historically severe underfunding by the government given the relative need of this population.^22^

During the COVID-19 pandemic, the higher rates of health challenges and lower rates of health insurance for individuals experiencing poverty contributed to worse health outcomes. Evidence shows that individuals in states without the Medicaid expansion were more likely to be at a higher risk for poor COVID-19 outcomes compared to states that accepted the Medicaid expansion; and these higher risk individuals were 52% more likely to be underinsured than comparable individuals in states with the Medicaid expansion.^20^ One contributing factor to these outcomes is that individuals lacking adequate health insurance are more likely to avoid healthcare services for preventative care or seemingly minor health problems compared to insured individuals.^18^ Even for those who seek healthcare services, the socioeconomic status of a patient can influence the quality of healthcare received, with lower income patients reporting poorer quality healthcare experience.^23^ According to the 2019 National Healthcare Quality and Disparities Report mandated by Congress, compared to White patients, Black and AIAN people received worse care on nearly 40% of healthcare quality measures and Hispanic and NHOPI patients received worse care on at least 30% of healthcare quality measures studied.^24^

Unequal vaccine distribution, particularly in the early months of the vaccine rollout, may have exacerbated COVID-related health disparities for lower-income and/or racial/ethnic minority individuals. Without health insurance, many individuals do not have access to a primary care provider and consequently lack a trusted source of vaccination information.^25^ The continued mistrust in the healthcare system by racial/ethnic minorities due to historical and ongoing mistreatment and systemic discrimination has correlated with vaccination hesitancy among minority populations, particularly Black and Hispanic patients.^26^ Racial/ethnic minorities are also less likely to receive adequate information regarding vaccinations. By April 2021, when the COVID-19 vaccination was available to all U.S. adults, 42% of unvaccinated Hispanic adults (compared to 30% of overall population) did not know if they were eligible to receive the COVID-19 vaccination, and 45% of unvaccinated Hispanic adults and 30% of Black adults (compared to 26% of overall population) did not have adequate information about where to receive a vaccination.^27^

The third mechanism connecting poverty and discrimination to COVID-19-related health outcomes relates to **lower-quality neighbourhood and housing conditions**. This includes neighbourhood characteristics (such as underemployment, underresourced schools, and more), as well as conditions of more specific living arrangements, such as number of household members or characteristics of community living environments.

At the neighbourhood level, economic and racial residential segregation has been associated with a more severe COVID-19 impact on lower income and racial/ethnic minority populations. Lower-income and racially segregated counties experience a significantly greater number of COVID-19 cases and deaths compared to higher-income and less segregated counties.^28^ In particular, COVID-19 cases and related deaths are highest in metropolitan counties with prevalent Black-White and Hispanic-White residential segregation, and these effects are further increased by large income inequality.^29^ A study focused on the U.S. epicenter of the pandemic, New York City (NYC), found that the Bronx had the most COVID-19-related hospitalizations and deaths, as well as the largest racial/ethnic minority and impoverished populations, of the five boroughs in NYC.^30^

The disparities are not limited to high-density cities, however. When focusing on suburban areas, U.S. counties where the majority of residents are AIAN have the highest COVID-19-related mortality rates.^31^ Black majority suburban and rural counties, as well as Hispanic majority rural counties, were found to have higher COVID-19 mortality rates compared to White majority equivalents.^31^

An analysis of micro-level housing factors further demonstrates that low-income and racial/ethnic minority individuals are at increased risk and vulnerability for COVID-19. With COVID-19 being a highly infectious disease, housing quality factors such as sanitation, plumbing, and overcrowding greatly impact an individual's vulnerability to contracting COVID-19. Households with a high housing cost burden (>50% of household income used to pay for housing cost), incomplete kitchen or plumbing facilities, and/or overcrowding were shown to have more COVID-19 cases and related deaths.^32^ Counties with a greater proportion of households with the previously outlined characteristics are more likely to have lower median household incomes, greater population density, and a larger share of racial/ethnic minority residents.^32^ Across the U.S., AIAN, Black, and Hispanic households are most likely to have incomplete household plumbing.^33^ Multigenerational households and overcrowded households are also associated with greater risk for COVID-19.^34^ According to the U.S. Census, households of color are more likely to be multigenerational compared to White households.^35^ Households identified as being overcrowded are more likely to be racial/ethnic minority and low income families.^36^ More specifically, 26.0% of Hispanic, 17.2% of Asian, and 14.4% of Black families, compared to only 10.1% of White families, live in households with five or more individuals.^37^ For AIAN households, 16% in tribal areas and 10% in urban areas are classified as overcrowded, compared to only 2% of all U.S. households.^38^

Overcrowding is also a major concern for areas of communal living, including homeless shelters, immigration detention centers, and prisons. Homeless shelters often have communal sleeping areas and shared hygienic facilities. Due to these suboptimal living conditions, COVID-19 can spread rapidly among residents and staff, as well as within surrounding communities. Case studies of homeless shelters in King County, Washington, have shown the rapid spread of COVID-19 within these shelters and demonstrated the importance of extensive testing programs.^39^ For any homeless individual unable to stay in a shelter, there are similar risks and concerns related to COVID-19. Primarily, these individuals are unable to shelter-in-place and may lack sanitation and health-related resources in their place of living.^40^ According to an analysis of 2020 data by the National Alliance to End Homelessness, NHOPI, Black, AIAN, Hispanic, and multiracial individuals are between 2 to 10 times more likely to experience homelessness compared to White counterparts.^41^ Similar concerns exist for immigration detention centers, which are often overcrowded, lack adequate sanitation measures, and under-resourced with personal protective equipment.^42^ The majority of detainees in U.S. immigration detention centers are from Mexico (43%) or Central America (46%), demonstrating the potential for racial/ethnic inequality in COVID-19 impact in detention centers.^43^

There is also evidence that renter evictions during the COVID-19 pandemic are aggravating the spread and impact of COVID-19, particularly for lower income individuals. Even before the COVID-19 pandemic, renters had, on average, fewer savings than mortgage holders, with 47.5% of U.S. renters spending more than 30% of their income on housing costs in 2018.^44^ Disruptions to rental stability, specifically evictions, therefore are likely to disproportionately affect low-income individuals. At the beginning of the COVID-19 pandemic, 43 states and the District of Columbia issued temporary eviction moratoria (with a median duration of 9.9 weeks); but the expiration of these moratoria was associated with increased COVID-19 cases and mortality rates.^45^ A national survey found that evictions during the COVID-19 pandemic have disproportionately affected Black and Hispanic populations, with these inequalities particularly impacting lower income Black and Hispanic households.^46^ Another study found that 78% of evictions in Boston, Massachusetts during the COVID-1-9 pandemic impacted individuals residing in census tracts where the majority of the population were racial/ethnic minorities.^47^ Not until September 2020, more than six months after the onset of the pandemic, was a national moratorium on evictions established.^48^

A less direct community-level factor that impacts COVID-19 vulnerability and is closely tied to income and race/ethnicity is access to COVID-19 testing. The CDC has indicated the importance of equitable access to COVID-19 testing across the nation. Adequate testing is necessary to identify infected individuals and to ensure they are able to seek early necessary healthcare to reduce severe COVID-19 outcomes and mitigate the spread of infection to others.^49^

Although the CDC has emphasized the importance of COVID-19 testing among the most vulnerable communities and populations, research indicates that there are large socioeconomic and racial/ethnic inequities in COVID-19 testing access. For example, U.S. states with higher poverty rates and greater shares of Black individuals tend to have lower COVID-19 testing rates.^50^ Specifically, states with the highest share of residents experiencing poverty (top quartile) had COVID-19 testing rates that were nearly 40% lower than testing rates in states with the lowest share of residents experiencing poverty (229.3 vs 375.2 per 100,000 residents). Similarly, states with the highest share of Black residents had COVID-19 testing rates that were nearly half of rates in states with the lowest share of Black residents (206.4 vs 403.5 per 100,000 residents). This data suggests that COVID-19 testing is not *equal*, let alone equitable, across socioeconomic and racial/ethnic divides. To further support this statement, a NYC-focused study found that, with increasing socioeconomic status (SES) index score and increasing share of White individuals, the ratio of positive COVID-19 tests to total performed tests decreased.^51^ In fact, one study found that the lowest-income neighbourhoods had a 62% positive testing rate, while the highest-income neighbourhoods had only a 35% positive testing rate.^52^ For low-income and racial/ethnic minority populations, a lack of adequate testing may exacerbate the increased vulnerability to COVID-19 infection and severe outcomes that already exist.

Neighbourhood and housing inequality that make low-income individuals more vulnerable to COVID-19 are similarly apparent when considering COVID-19 vaccinations. Primarily, housing status, residential segregation, and limited household resources create barriers for low-income and racial/ethnic minority populations from accessing the COVID-19 vaccination. A study at Princeton University found that zip codes with higher eviction rates generally had lower vaccination rates.^53^ Other researchers found that, in 94 U.S. counties, Black residents were more likely than White counterparts to have to travel more than ten miles to a vaccination center.^54^ Other household characteristics that impact one's access to a COVID-19 vaccination include technological and transportation resources. Due to the use of online appointment scheduling, individuals lacking adequate internet access or technological resources (who are primarily lower-income households) were more likely to experience barriers to COVID-19 vaccination.^55^ A lack of adequate internet access is particularly prevalent in communities where more racial/ethnic minorities live. ^56^ For many individuals, particularly low-income, Black, and Hispanic individuals, transportation may also be a barrier to acquiring the vaccination.^57^

The fourth mechanism connecting poverty to COVID-related health outcomes pertains to **unequal reliance on employment in high-risk occupations**. Prior to the onset of the pandemic, the federal minimum wage was $7.25 per hour (unchanged since 2009), union membership was low, and the out-of-work safety net had been gradually weakened.^1^^,^^58^ As a result, a large share of working-age individuals worked in low-pay and/or low-quality jobs. Amidst the pandemic, many of these jobs became hazardous, with little escape for workers unable to perform their duties remotely.

“Essential workers”, in particular, are consistently shown to be at increased risk for COVID-19 infection and death. As defined by the CDC, essential workers are employed in occupations that are necessary “to ensure the continuity of critical functions of the U.S.”^59^ This includes healthcare workers and non-health related workers who are employed in sectors such as education, critical retail, public transportation, and food production. In each U.S. state, at least 39% of individuals in the general workforce are essential workers.^60^ The increased COVID-19 risk of essential workers is, in part, due to their inability to work remotely, but also due to inadequate working conditions and health regulations. Essential workers have reported working conditions that do not follow health guidelines, such as a lack of social distancing, insufficient personal protective equipment provisions, and inadequate facilities to maintain personal hygiene and sanitation.^61^

A significant share of U.S. frontline and essential workers are low-income and racial/ethnic minorities. Almost one-quarter of frontline workers are low-income, as defined by living at or below twice the federal poverty level.^62^ A NYC-specific study reported similar findings, with 8% of frontline workers living at or below the federal poverty level and 24% of frontline workers living at or below twice the federal poverty level.^63^ Correlates for income support these findings, with 8% of NYC frontline workers lacking healthcare coverage (comparable to the 8% of individuals in the U.S. lacking health insurance coverage in 2019)^64^ and with frontline workers being more likely to rent, rather than own, a home.^63^ Additionally, workers in the bottom decile for work salary were almost three times more likely than workers in the top quartile to lack paid sick leave, potentially adding to the increased vulnerability of disproportionately low-income frontline workers.^65^ There is also an overrepresentation of racial/ethnic minority workers in essential occupations.^62^ Notably, racial/ethnic minority workers make up 75% of all NYC frontline workers, with greater than 50% of NYC frontline workers being immigrants.^63^ These individuals, notably, faced reduced access to COVID-19-related income support during the initial months of the pandemic, increasing their reliance on earnings from employment to maintain financial stability.^66^

Research has shown that essential workers, and therefore a disproportionate share of racial/ethnic minority workers, are at increased risk for COVID-19 infection and death. A study on industry sector workplace COVID-19 outbreaks in Utah showed that 73% of outbreaks affected Hispanic or non-White (including Black, AIAN, Asian, and NHOPI) individuals, a disproportionate impact considering that less than 24% of workers in the affected occupation sectors were Hispanic or non-White.^67^ In state analyses of essential workers in California and Massachusetts, there were greater COVID-19 mortality rates among Hispanic and Black workers.^68^^,^^69^ In fact, age-adjusted mortality rates among workers in Massachusetts were four times higher for Hispanic and Black high-risk occupation workers compared to White counterparts.^69^

Labour market inequalities may also affect income-related disparities in vaccine access. Despite being among the first individuals to have access to the vaccination, essential and frontline workers experienced many barriers to vaccination. For example, an inflexible work schedule may have made it difficult for many to schedule a vaccination appointment, especially in the first few months of vaccination distribution when appointments were sparse. Additionally, lower-income workers may face more difficulty in taking time off work to receive the vaccine or in case of experiencing adverse side effects post-vaccination. Not until mid-March 2021 did states, such as New York and California, begin to mandate paid leave for workers receiving the COVID-19 vaccination.^70^^,^^71^

## Future Research Needs on Poverty, Discrimination, and COVID-19 Health Disparities

Studies on the impact of COVID-19 on under-researched minority populations – such as AIAN, NHOPI, gender/sexual orientation minorities, and disabled individuals – would further enhance our understanding of the socioeconomic impact on COVID-19 vulnerability. These under-researched minority populations all tend to be socioeconomically disadvantaged, likely placing them at increased vulnerability for negative COVID-19 outcomes.^72^, ^73^, ^74^ Another area of research worthy of further investigation is the relative COVID-19 vulnerability of specifically low-income racial/ethnic minorities. This is particularly important for Asian individuals in the U.S. According to the Pew Research Center, the Asian population is the most economically divided racial/ethnic group in the U.S., with the top income decile earning, on average, 10.7 times the amount of the bottom income decile.^75^ Asian individuals at the top and middle of the income distribution tend to have the highest incomes relative to White, Black, and Hispanic counterparts; however, within the bottom decile, Asian individuals have a lower income than White counterparts.^75^ Many studies included in this Viewpoint grouped all Asian individuals, independent of income, into a single category. Without more research on specifically low-income Asian populations, there is the risk of ignoring potential disparities in COVID-19 vulnerability for low-income Asian individuals. Focused study of low-income individuals of other races/ethnicities will be beneficial in a similar manner, although other races/ethnicities are less economically divided compared to the Asian population.

## Conclusion

On the eve of the pandemic, the U.S. featured high poverty rates relative to other high-income countries. ^3^ The high rate of poverty, particularly among racial/ethnic minorities, exists hand-in-hand with historic racial discrimination and comparatively weak welfare state and labour market institutions in the U.S.^3^ Our review of currently available evidence suggests that these conditions exacerbated the health consequences of the COVID-19 pandemic, particularly for racial/ethnic minorities.

Specifically, the evidence suggests that poverty and discrimination affect COVID-19-related health consequences through the four primary mechanisms discussed above: greater pre-existing health challenges, reduced access to healthcare, lower-quality neighbourhood and housing conditions, and unequal reliance on employment in high-risk occupations. Our review of the evidence suggests that economic and policy institutions that contributed to higher pre-pandemic poverty rates in the U.S., particularly among racial/ethnic minorities, have been central determinants of unequal health outcomes during the COVID-19 pandemic.

## Declaration of Interests

There exist no conflicts of interest.
